# Characteristics of Solidified Carbon Dioxide and Perspectives for Its Sustainable Application in Sewage Sludge Management

**DOI:** 10.3390/ijms24032324

**Published:** 2023-01-24

**Authors:** Joanna Kazimierowicz, Marcin Dębowski

**Affiliations:** 1 Department of Water Supply and Sewage Systems, Faculty of Civil Engineering and Environmental Sciences, Bialystok University of Technology, 15-351 Bialystok, Poland; 2 Department of Environment Engineering, Faculty of Geoengineering, University of Warmia and Mazury in Olsztyn, 10-720 Olsztyn, Poland

**Keywords:** anaerobic digestion, dewatering, pretreatment, sanitization, sewage sludge, solidified carbon dioxide

## Abstract

Appropriate management is necessary to mitigate the environmental impacts of wastewater sludge. One lesser-known technology concerns the use of solidified CO_2_ for dewatering, sanitization, and digestion improvement. Solidified CO_2_ is a normal byproduct of natural gas treatment processes and can also be produced by dedicated biogas upgrading technologies. The way solidified CO_2_ is sourced is fully in line with the principles of the circular economy and carbon dioxide mitigation. The aim of this review is to summarize the current state of knowledge on the production and application of solid CO_2_ in the pretreatment and management of sewage sludge. Using solidified CO_2_ for sludge conditioning causes effective lysis of microbial cells, which destroys activated sludge flocs, promotes biomass fragmentation, facilitates efficient dispersion of molecular associations, modifies cell morphology, and denatures macromolecules. Solidified CO_2_ can be used as an attractive tool to sanitize and dewater sludge and as a pretreatment technology to improve methane digestion and fermentative hydrogen production. Furthermore, it can also be incorporated into a closed CO_2_ cycle of biogas production–biogas upgrading–solidified CO_2_ production–sludge disintegration–digestion–biogas production. This feature not only bolsters the technology’s capacity to improve the performance and cost-effectiveness of digestion processes, but can also help reduce atmospheric CO_2_ emissions, a crucial advantage in terms of environment protection. This new approach to solidified CO_2_ generation and application largely counteracts previous limitations, which are mainly related to the low cost-effectiveness of the production process.

## 1. Introduction

The activated sludge process, which is currently the most widespread method of wastewater treatment, is irrevocably linked to a high production of sludge that cannot be directly and safely neutralized in the environment due to its quality and characteristics [1]. Appropriate sludge management is necessary to reduce its environmental impacts. The sewage sludge generated during wastewater treatment must be processed (often in multistep processes), then properly neutralized and/or managed [2]. Despite the great strides in developing techniques and methods of sludge management, a universal, economically viable technology for its effective neutralization has yet to be developed [3]. This is a pressing issue, as more and more sludge is being produced. The global trends and dynamics of wastewater treatment system development give reason to believe that the scale of the problem will grow further still.

**Figure 1:**
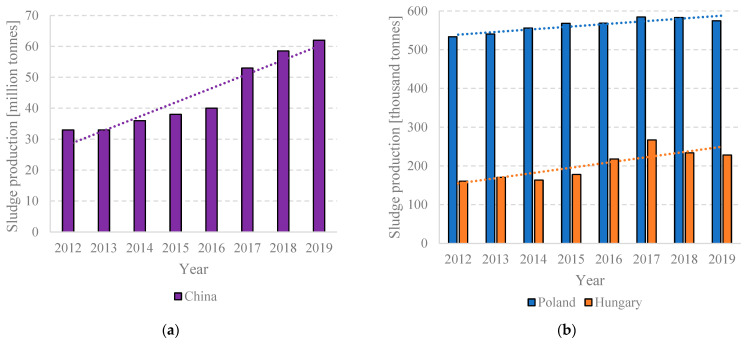

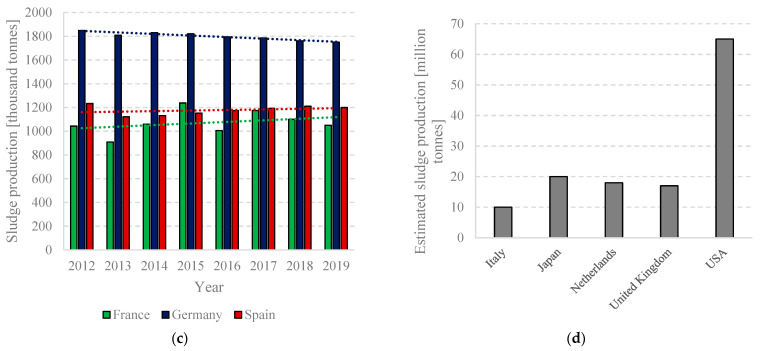
Sludge production (**a**) increase in developing countries (Poland and Hungary), (**b**) China; (**c**) in developed countries (France, Germany and Spain); and (**d**) in selected countries.

According to UNESCO’s World Water Assessment Programme (WWAP) [4] and United Nations Water [5], over 80% of the sludge generated worldwide is still discharged directly into the environment, a statistic that rises to 95% in developing countries. Sustained deployment of efficient wastewater treatment systems will inevitably increase the amounts of sludge generated, which, given its impact on the environment, will need to be neutralized and managed [6]. Sludge is detrimental to the environment due to its susceptibility to putrification, sanitary issues, nuisance smells, and release of aerosols/micropollutants [7]. For this reason, issues of wastewater and sludge management have been included in the Agenda for Sustainable Development 2030 via Sustainable Development Goal (SDG) 6, which concerns developing technologies for wastewater treatment and safe water/sludge recycling [8,9]. The current annual sludge generation is estimated at 150 million tons of dry mass in Europe, 30 million tons of dry mass in China, and approx. 65 million tons of dry mass in the United States and is expected to grow even further as economies and populations grow [10]. A particularly significant increase in sewage sludge production is observed in rapidly developing countries and emerging economies. These include most Asian countries, especially China (Figure 1a) [11], and South American countries, while in Europe, these are countries that joined the EU structures after 2004, including Poland and Hungary (Figure 1b) [12]. A similar situation will most likely be observed in the future on the African continent. This is directly related to the economic development of African countries, growing national income, increasing environmental awareness, and the need to adapt to global agreements on sustainable development. Therefore, this affects the dynamic development of wastewater treatment systems and thus causes an increase in the amount of sewage sludge. In economically developed countries where wastewater management is well implemented, such as in France and Germany (Figure 1c), the amount of sewage sludge produced has usually stabilized at a high, constant level for many years [12]. Sludge generation in selected countries is given in Figure 1d. 

Many different technologies are used to manage and neutralize sewage sludge, with varying levels of sophistication, complexity, investment/operating costs, environmental outcomes, and end products obtained [13]. Well-explored, well-known, and practiced sludge processing methods include gravity and mechanical thickening, [14], dewatering in natural or controlled conditions [15], desiccation [16], landfilling [17], natural or agricultural use [18], thermal treatment (including combustion, gasification, pyrolysis, and plasma technology) [19], biological/chemical stabilization [20], biodegradation through composting [21], anaerobic digestion (AD) [22], and fermentative hydrogen production [23]. 

Options of disposing sludge by landfilling or immediate reuse in agriculture or forestry are becoming more and more limited due to the high pollutant content and sanitary risk. The same applies to using sludge for reclaiming impoverished or degraded soil [24]. Multiple countries have legally banned these particular methods of sludge control [25]. Combusting sludge is expensive and poses a risk of releasing toxic substances, such as dioxins, furans, and heavy metals [26]. One environmentally friendly alternative and competition to incineration lies in low-oxygen processes, gasification, and pyrolysis, but such installations and technologies usually have a low technology readiness level (TRL) [27]. Composting is a bioconversion technology that aims to transform sewage sludge into organic fertilizer (to recycle mineral elements and organic matter for soil amendment) [28].

Compared to these methods of conditioning and managing sewage sludge, AD emerges as a technologically profitable and environmentally friendly solution [29]. When performed correctly, it lowers the putrification susceptibility of the sludge, removes biodegradable organic matter, partially sanitizes the substance, reduces sludge volume, and produces methane- or hydrogen-rich biogas [30]. AD enables energy capture producing biogas that can be used as a source of heat, electricity or, after upgrading, directly as fuel for compression ignition engines [31]. 

In the current geopolitical and environmental climate, energy diversification and biofuel production from renewable sources are high priorities. The gravity of the problem stems from multiple factors, including the lack of geographic equity in access to energy carriers; the use of energy resources as a political weapon; environmental risks of greenhouse gas emissions from conventional energy sources; political, military, and environmental consequences of nuclear programs; and risk of energy dependence and the associated knock-on effects [32]. The military conflict in Ukraine has exacerbated the energy crisis and pushed the importance of pursuing energy independence to global attention. Countermeasures need to be taken to tackle these issues, including diversifying energy sources, employing dispersed systems, and improving local fuel production capacities. 

Biogas produced via AD of various feedstocks, including sewage sludge, has been recognized under EU Directive 2009/28/EC (the ‘Energy Directive’) as a renewable energy source, one which can significantly improve the share of renewable energy in the energy mix, thus potentially helping deal with the ongoing energy crisis and preventing similar ones in the future [33]. This view is justified by an in-depth understanding of AD in terms of the taxonomic structure of the anaerobic microorganisms, specifics of biochemical conversions, chemical reactions, technological parameters, aspects of design, construction and operation, and extensive knowledge on how to handle biogas and where to best use it, gained from previous research [34,35].

One important segment of research that needs to be explored further is pilot-scale and full-scale experiments/studies aiming to develop successful and cost-efficient methods of pretreating organic feedstock for AD [36]. Pre-AD sludge disintegration methods are a fast-growing technology [37]. They are used to disrupt the structure of the sludge by separating flocs, destroying microbial cells, dissolving organic matter and extracellular polymers, etc. [38]. There have been reports in the literature on many different methods of disintegration, including those based on mechanical treatment [39], high-pressure treatment [40], soundwaves [41,42], microwaves [43,44], cavitators [45], and biological methods [46] and chemical methods, such as acidification [47], alkalization [48], oxidation [49], ozonation [50,51], as well as thermal methods, such as heat treatment [52] and freezing/thawing [53]. These individual disintegration methods can also be combined for a hybrid approach [54,55].

Most of the tested pretreatment methods are effective in facilitating AD [56]. When implemented, they can significantly improve biogas production [57], methane and/or hydrogen fractions [58], mineralization, and organic matter removal [59]. However, using pre-AD sludge disintegration methods usually results in a negative energy balance, meaning that the net energy produced does not offset the input required to run the pretreatment systems [60]. Ample studies have also pointed to the high investment costs and the costs of servicing, maintenance, and repair of disintegration systems [61]. Processes based on advanced technologies require qualified service technicians and are highly complex, which frequently goes hand in hand with operational hurdles and markedly reduced output [62]. Given these considerations, it is necessary to identify new, versatile, and environmentally friendly technologies for sewage sludge pretreatment, ones which could serve as a viable alternative in terms of cost-effectiveness and AD performance [63]. 

One interesting and promising proposal calls for using solid carbon dioxide (SCO_2_) to process sewage sludge. Solid CO_2_ is a normal byproduct of natural gas treatment processes and can also be produced by dedicated biogas upgrading technologies [64]. Given the origin and sourcing of SCO_2_, this method could be considered material recycling and is fully in line with the principles of the circular economy [65]. It can also help limit carbon dioxide emissions by sequestering and feeding it into a closed-loop process [66]. Methods of producing and harnessing SCO_2_ for sludge disintegration encompass the capture, extraction, transport, and long-term storage of CO_2_ in a suitable and safe location [67]. To date, little information has been reported in the world literature regarding the feasibility of the low-temperature conditioning of surplus sludge using solidified carbon dioxide (LTC-SCO_2_). As such, it is still a relatively nascent technology.

The present review article aims to summarize the current state of knowledge on the production and applications of SCO_2_ in pre-AD processing and the pretreatment of sewage sludge. It puts special emphasis on assessing how this process changes sludge properties and how disintegration affects the final performance in terms of dewatering, sanitization, and digestion efficiency. The literature review serves as a basis to evaluate the competitiveness of this technology and to consider further research and actions aimed at determining whether the technology is viable in full-scale plants.

## 2. Characteristics of Sludge

**Figure 2:**
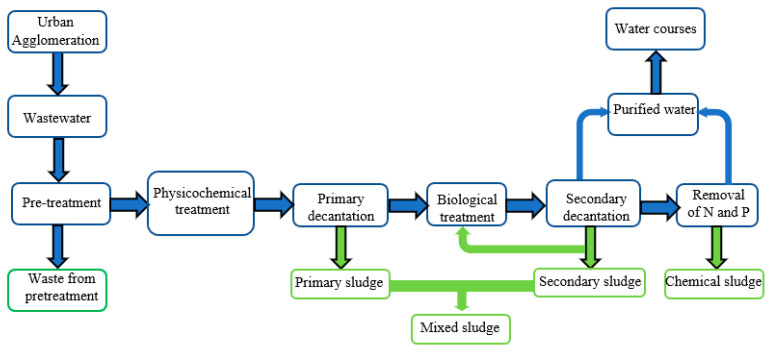
Biological wastewater treatment plant with separate sludge generation subprocesses. N—nitrogen; and P—phosphorus.

Sludge is a byproduct of wastewater treatment processes, and its quality is directly tied to its composition and characteristics [68]. It is a biological community composed of microbes, nondegraded organic matter from sewage, multiple mineral pollutants, and water [69]. Sludges are classified according to their origin and production process [70]. One of the types is primary sludge generated via gravity sedimentation of readily settleable suspensions in primary sedimentation tanks [71]. The second commonly cited type is secondary (surplus) sludge, which is the active sludge biomass settled out in secondary sedimentation tanks, grown in the process of sewage biotreatment [72]. The third type is chemical sludge, often formed during the chemical precipitation of biogenic substances (usually phosphorus removal with inorganic coagulants and polyelectrolytes) [73]. It is common practice at municipal and industrial wastewater treatment plants to converge all streams of sewage sludge into a mixed sludge for further processing, neutralization, and management [74]. A flowchart of a typical biological wastewater treatment plant (with separate subprocesses for sludge generation) is shown in Figure 2. 

It is estimated that sewage sludge management and disposal generates approx. 50% of the operating costs of wastewater treatment plants [75]. Sludge separated by gravity in sedimentation tanks has a high water content between 97 and 99%. Before being fed into digesters, the raw sludge is usually dewatered mechanically, often using added polyelectrolytes [76]. The dewatering produces organic feedstock with 87–95% water content [77].

The best and most cost-effective strategy, in line with the principles of sustainable development and the circular economy, is to reclaim and reuse sludge as fertilizer [78]. After all, sludge is a widely available, easily accessible, and inexpensive source of nutrients, including nitrogen, phosphorus, and structure-forming organic matter [79]. Sewage sludge also contains yield-enhancing micronutrients, including copper, zinc, molybdenum, boron, iron, magnesium, and calcium [80]. Using high-quality sewage sludge on low-quality agricultural land can improve the physical, chemical, and biological properties of the soil [81]. Sludge-amended soils have shown increased microbial counts, and with them, increased respiration and enzymatic activities [82]. The possibility of environmental or agricultural use of sewage sludge is determined by its characteristics and composition. Important indicators include the content of nutrients and fertilizers, concentrations of heavy metals and other nonspecific pollutants, including micropollutants, as well as the value of sanitary indicators. The values of these parameters, which are important from the point of view of the final neutralization of sewage sludge, are influenced by the origin of this waste, but also by the sludge management methods used, including dewatering, disinfection, pretreatment, or anaerobic digestion, i.e., the processes for which SCO_2_ can be used. The characteristics and composition of sewage sludges are presented in Table 1. 

The suitability of sludge for disposal and reuse in the environment is largely determined by its content of heavy metals and its sanitary indicators [94]. The heavy metal content of sludge, including Cu, Ni, Zn, Cr, Cd, Mn, and Pb, can range from 0.5 to 2% dry matter, or even up to 6% in some cases [86]. If the sludge contains high amounts of heavy metals, these metals may potentially be released into the environment and enter the food chain [95]. The concentrations of heavy metals in sewage sludge are given in Table 2. The types of pathogenic organisms prevalent in sludges and derived products (compost, desiccated sludge, stabilized sludge, anaerobically digested sludge, etc.) are a function of the facilities and conditions present in the area that produced the treated wastewater, namely public health, hospitals, tanneries, meat establishments, and slaughterhouses [96]. Despite the high sanitary standards of developed countries, the degree of pathogenesis and prevalence of pathogenic microorganisms is usually significant. Currently, the sanitary quality of sludge is assessed based on the presence (or lack thereof) of *Salmonella* sp. and live eggs of the intestinal worms *Ascaris* sp., *Trichuris* sp., and *Toxocara* sp. [97]. Sanitary indicators for sludge from municipal wastewater treatment plants are presented in Table 3. 

Organic micropollutants are now considered to play an important role due to their carcinogenic and mutagenic properties (Table 4). The reuse of sludge with high concentrations of such substances, whether in the environment or otherwise, can pose a direct risk to human health [111]. The types of organic micropollutants most prevalent in sludges are polycyclic aromatic hydrocarbons (PAHs), pharmaceuticals (PhCs), polychlorinated biphenyls (PCBs), perfluorocarbons (PFCs), per- and polyfluoroalkyl substances (PFASs), benzotriazoles, nanoparticles, pesticides, and surfactants [112]. It has been proven that the AD process can reduce these types of impurities. Abril et al. (2020) [113] demonstrated a reduction in the content of anionic surfactants during the AD of sewage sludge. In the research of Phan et al. (2018) [114], the mass balance showed that during AD, the biotransformation was significant for six hydrophilic PhC compounds, namely atenolol, caffeine, trimethoprim, paracetamol, naproxen, and sulfamethoxazole. Gonzalez-Gil et al. (2016) [115] also found a high efficiency of 85% removal of sulfamethoxazole during the mesophilic (37 °C) and thermophilic (55 °C) AD of sewage sludge. Li et al. (2021) [116] obtained the degradation of polyfluoroalkyl phosphates during the AD of sewage sludge. However, the researchers emphasize that the ability of AD to remove organic micropollutants by biotransformation is limited, which can be improved by using pretreatment techniques. Pretreatment processes, including the use of SCO_2_, may be of significant importance for the degradation of sequestered or highly hydrophobic compounds, mainly through their transition to the soluble phase and then increased bioavailability. For example, Braguglia et al. (2015) [117] evaluated the effect of AD combined with pretreatment in the form of thermal hydrolysis and ultrasound on the concentration of micropollutants in sewage sludge. The PAH biotransformations ranged from 33 to 75%, while PCBs were 70% [117]. 

Sludges are increasingly being utilized as precursors of energy carriers [123,124]. The calorific value of dry organic constituents of sludge ranges between 18 and 21.5 MJ/kg. By comparison, the ranges for traditional fuels are 21–25 MJ/kg for hard coal, 45 MJ/kg for light fuel oil, and 48 MJ/kg for natural gas [125]. Processes used to extract energy from sludge include burning after desiccation [126], coincineration with coal or other biomass [127], low-oxygen thermal treatments including pyrolysis and gasification [89], and plasma technologies [128]. Sludges are also often bioconverted to gas energy carriers via anaerobic processes, including AD and fermentative hydrogen production [129,130].

## 3. SCO_2_ Characteristics, Production Methods, and Applications

**Figure 3:**
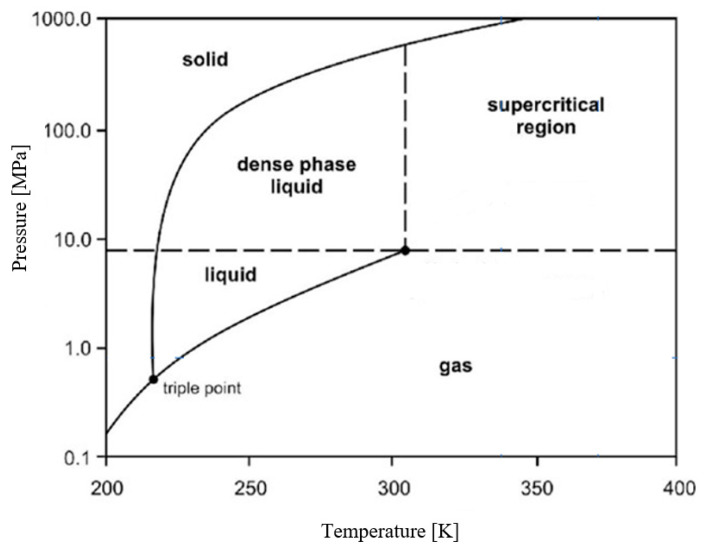
Relationship between phase change of CO_2_ and temperature/pressure [132].

Solid carbon dioxide (SCO_2_) is the solid state of CO_2_, a molecule composed of a single carbon atom bonded to two oxygen atoms. At pressures below 5.13 atm and temperatures exceeding −56.4 °C (the triple point), CO_2_ turns directly from a solid into a gas without going through the liquid phase, a process known as sublimation [131]. The opposite of sublimation is deposition, which is when CO_2_ transitions from a gas to a solid (dry ice). Under atmospheric air pressure, sublimation/deposition occurs at −78.5 °C (sublimation enthalpy = 573 kJ/kg), making dry ice 3.3 times more efficient than water ice (volume-for-volume) [132]. The relationship between phase changes of CO_2_ and temperature/pressure is illustrated in Figure 3 [132]. 

The specific gravity of SCO_2_ ranges from 1.2 to 1.6 kg/dm^3^, with a Mohs hardness of 2 (equivalent to gypsum) [133]. SCO_2_ is noncombustible, odorless, tasteless, and nonpoisonous. When dissolved in water, it can lower the pH of the solution, forming carbolic acid (H_2_CO_3_) [134]. The low temperature of SCO_2_ and its ability to sublimate directly into gas make it a good coolant because it is colder than water ice and not prone to leaving residues during phase transition [135]. As SCO_2_ is apolar and has a dipole moment of zero, and it is associated with the emergence of van der Waals forces (intermolecular attractive forces) [136]. Its thermal conductivity and electrical conductivity are low due to its composition [137]. It is generally accepted that SCO_2_ was first observed in 1835 by the French inventor Adrien-Jean-Pierre Thilorier (1790–1844), who was also the first to publish a description of the substance [138]. In 1924, Thomas B. Slate filed for a US patent for a method of producing SCO_2_, then commercialized the production and marketed the substance [139]. In 1925, the invented name of this solid form of CO_2_ was registered as “dry ice” by the Dry Ice Corporation of America [140]. That same year, the substance found use in refrigeration [140].

SCO_2_ has also been found to occur in nature, for example, in the ice caps and dry ice storms over Mars [141]. In 2012, the European Space Agency probe Venus Express detected a cold layer of the Venusian atmosphere, where temperatures are close to the triple point of carbon dioxide and SCO_2_ flakes can form naturally [142]. Observations of Uranus by Voyager 2 indicate that SCO_2_ can also occur on the surface of its larger moons, Ariel, Umbriel, and Titania [143]. 

**Figure 4:**
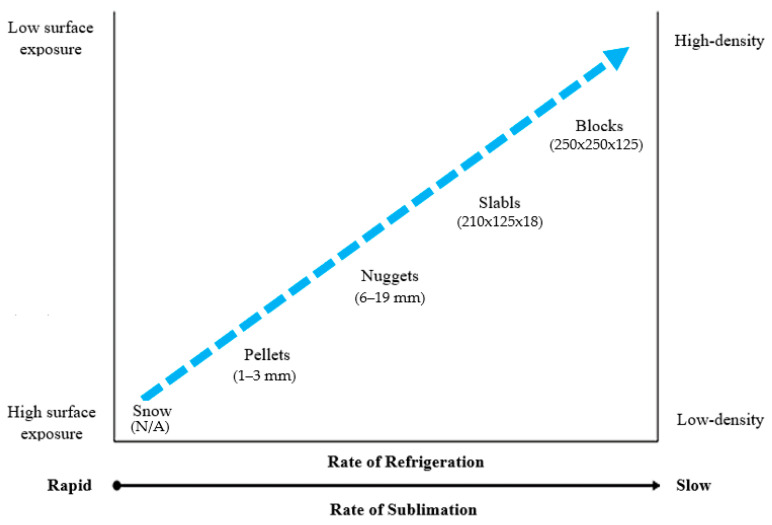
Classification of SCO_2_ types according to size, shape, active surface area, and sublimation rate.

SCO_2_ is relatively simple to produce. The process starts with gases rich in carbon dioxide [144]. The CO_2_-rich gas is compressed and cooled until liquefied. This pressure is then reduced, causing a portion of the liquid carbon dioxide to evaporate, thus drastically lowering the temperature of the remaining liquid. In this extreme cold, the liquid solidifies into a snow-like consistency. The final step is to pack the solidified carbon dioxide “snow” into smaller nuggets or larger blocks of SCO_2_ [145]. Most of the SCO_2_ is produced in one of the three standard forms: large blocks, small cylindrical nuggets (1/2 or 5/8 inches; 13 or 16 mm diameter), or pellets (1/8 inches; 3.2 mm diameter) with a high specific surface area [146]. Other intermediate forms are also in use. SCO_2_ pellets are mainly used for flash freezing, fire extinguishing, and oil solidification. They are also deemed safe for experimentation in junior high schools, as long as suitable personal protective equipment is worn, such as gloves and safety goggles. The blocks tend to be around 30 kg in weight and are used in maritime transport due to their slow sublimation rate (a product of their low surface-to-volume ratio). The nuggets are approx. 1 cm (0.4 inches) in diameter and are easy to pack. This form is suitable for small-scale use, such as in grocery shops and laboratories, where it is stored in a thick, insulated box [147]. The density of pellets is usually 60–70% that of blocks [148,149]. A classification of the SCO_2_ types according to size, shape, active surface area, and sublimation rate is shown in Figure 4, with a more detailed characterization given in Table 5.

SCO_2_ creates a bacteriostatic environment that ensures the quality of the refrigerated products by preventing oxidization. It is used in gastronomy, for refrigeration, for cleaning of various machinery, for slowing down exothermic reactions in laboratories, and recently for treating sewage sludge [151,152]. The substance has garnered widespread use, because it does not have a liquid phase and sublimates directly from a solid to a gaseous state at atmospheric pressure. While mostly employed as a coolant, it is also used in theatrical smoke and fog machines for dramatic effect. The advantages of dry ice are that it is colder than water ice and leaves no residue (apart from incidental frost from the moisture in the air). It is useful for storing frozen foods (such as ice cream) when mechanical refrigeration is not available. The most common application of SCO_2_ is in preserving food with noncyclical refrigeration [153]. It is often used to package items that need to remain cold or frozen, such as ice cream or biological samples, when mechanical cooling is not available or possible [135]. 

SCO_2_ is key in deploying vaccines, which must be stored at extremely low temperatures across the supply lines [154]. It can also be used to flash-freeze foodstuffs [155], laboratory samples [156], and carbonated drinks [157]; to produce ice cream [158]; and to solidify oil spills [159]. In laboratories, it is used to run cold chemical reactions and condense solvents [160]. Dry ice is used to arrest insect activity in closed containers of grains and cereal products, as it displaces oxygen, but does not alter the taste or quality of the food [161]. This also makes it a useful tool for preventing or delaying rancidification of food oils and fats [162]. Placing SCO_2_ in water accelerates sublimation and leads to the formation of a thick, smoke-like haze that sticks close to the floor. This property is utilized by smoke/fog machines for theaters and nightclubs [135,163]. Plumbers use equipment that feeds pressurized liquid CO_2_ into pipes; the SCO_2_ freezes the water and forms an ice plug, allowing repairs to be made without shutting down the water mains [164]. It is used as a bait to catch mosquitoes, bedbugs, and other insects attracted to carbon dioxide [165]. SCO_2_ has also found use in rodent control; dry ice pellets are dropped into rodent burrows, and the exit is cut off. This serves to asphyxiate the critters as the SCO_2_ sublimates [166]. Tiny pellets of SCO_2_ can be used to extinguish fires by cooling fuel and/or snuffing out the fire by removing the oxygen supply [167]. Under low temperatures, viscoelastic materials transition into the glass phase, making the substance useful for removing various types of pressure-sensitive adhesives, floor tiles, or plating/sheathing materials [168]. SCO_2_ has also found application in the assembly of cylinder liners for large engines; the liner is cooled so that it shrinks and slides freely into the engine block. Similar procedures are used in the manufacture of highly resilient mechanical assemblies, eschewing the need for studs, mortices, or welds [169].

One of the major mechanical applications of SCO_2_ is in blast cleaning, where SCO_2_ pellets are propelled from a compressed air nozzle, combining the high blasting speed with sublimation action [170]. This helps avoid leaving residues and soling surfaces, such as ink, glue, oil, paint, mold, and rubber. SCO_2_ blasting can be used in lieu of sanding, steam sanding, wet sanding, or solvent sanding. SCO_2_ blasting does not leave much residue in the environment apart from the sublimating CO_2_, making it an environmentally friendly technique [171]. SCO_2_ can also be used for removing flammable vapors from storage tanks; the sublimation of the SCO_2_ pellets within an evacuated and ventilated tank produces a burst of CO_2_ that carries the flammable vapors with it [172]. Current applications of SCO_2_ are listed in Table 5. 

Long-term exposure to SCO_2_ may cause severe damage to the skin from frostbite. SCO_2_ sublimates into large volumes of gaseous carbon dioxide, creating a hypercapnia hazard and should only be exposed to open air in well-ventilated areas [173]. For this reason, for the purposes of laboratory safety, the substance carries the warning label: “Store in a well-ventilated place.” Industrial SCO_2_ may contain contaminants that render it unsafe for direct contact with food [174]. SCO_2_ is not classified as a dangerous substance by the European Union [175] nor as a dangerous material for land transport by the US Department of Transportation. However, it is regulated as dangerous for the purposes of air and maritime transport, requiring compliance with IATA Packing Instruction 954 (IATA PI 954) and specific marking, including a UN 1845 black-and-white diamond sticker. Proper ventilation must also be maintained so that the packaging does not burst under increased pressure [176]. The US Federal Aviation Administration allows airline passengers to carry up to 2.5 kg per person in hold or hand baggage for storing perishable foods [140,177].

## 4. Production of SCO_2_ in Flue Gas Treatments and Biogas Upgrading Processes 

Extensive carbon dioxide (CO_2_) emissions into the atmosphere are one of the drivers of global warming, thus contributing to an environmental crisis that is having widespread repercussions [178]. It is estimated that CO_2_ accounts for almost 65% of total greenhouse gas (GHG) emissions [179]. Mitigating the effects of climate change has become a serious issue for the global economy and energy/environmental policy over the past few decades [180]. Threats to humanity posed by climate and ecological disasters have forced drastic action toward reducing CO_2_ emissions [181]. European Union (EU) countries have taken bold and uncompromising action in this regard, successively and systematically implementing ambitious plans to achieve carbon neutrality by 2050 [182]. 

One applicable method of CO_2_ capture is cryogenic separation (distillation), where CO_2_ is condensed at low temperatures and at sufficiently high pressure, after which the separated components are removed in liquid or solid form [183]. This method for CO_2_ separation has been considered unattractive due to compression and other factors, but can prove beneficial when combined with other techniques, especially in situations where carbon dioxide has to be both purified and liquefied prior to transport [184]. CO_2_ extraction from flue gas requires between 0.6 and 1.0 KWh/kg CO_2_ [185]. This can also be combined with common techniques that trigger the phase transition of energy carriers for transport and use [186]. One example is the shipment of liquid natural gas (LNG) by sea. LNG is evaporated into gas at demand units/terminals, then injected into domestic pipe networks. The LNG phase change is an exoenergetic reaction that releases significant amounts of energy, which can be drawn from the surrounding environment. This is what is known as a ‘cold energy’ process, and can be used to cryogenically sequester and liquefy/solidify CO_2_ from flue gas [187]. Only solid CO_2_ is stable under atmospheric pressure, putting this product at an advantage. This technology is used by the Chinese Petroleum Corporation (CPC) [188]. 

Low-temperature CO_2_ removal from mixed gas is increasingly being applied to condition and upgrade biogas in the context of biomethane production [189]. Cryogenic technologies are an innovative set of techniques based on harnessing the fact that different constituents of gas mixtures (including crude biogas streams) have different condensation temperatures. Since SCO_2_ is stable under atmospheric conditions and has a variety of different applications, SCO_2_ production has become the preferred method of CO_2_ fixation and recovery from biogas for many researchers [190]. Biomethane can also be cooled down into liquid, in which form it can be distributed and spent via established LNG systems [66,191]. 

Liquid biomethane is a versatile, easy-to-transport energy carrier that can be stored for long periods of time on account of its significantly reduced volume (by a factor of approx. 1000 compared to biogas). Due to the high energy density of liquid biomethane (Hs = 6.44 kWh per liter, compared to approx. 0.0064 kWh per liter of biogas), it can be transported to an energy-efficient power plant just as easily as using fuel for trucks [192], with liquefied biomethane (LBM) trucks already widespread in the USA. The cold LBM could also find secondary use in food transportation to refrigerate cargo. The ‘by-product’ SCO_2_ is actually a value-added coproduct of the cryogenic process, which can be sold to various industries at high prices [193]. 

Recent years have seen the emergence of new and innovative ideas on how to implement small-scale biogas upgrading techniques to improve the energy efficiency of small agricultural biogas plants [194]. The concept of pressure-free cryogenic biogas conditioning/upgrading is based on bespoke upstream gas purification processes and subsequent low-temperature treatment, where the SCO_2_ is deposited first, then the remaining methane liquefied. Gas purification is associated with improving the technical functionality and the energy efficiency of the cryoprocess. Care must be taken to prevent impurities in the SCO_2_ mass, which may be detrimental to its marketability. During the gas separation, the focus is on stable, reliable, and always-on heat exchangers. 

**Figure 5:**
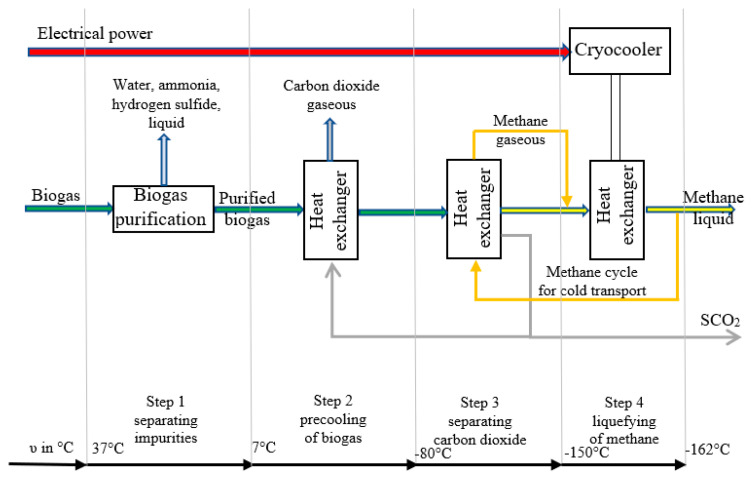
Biogas upgrading with concurrent SCO_2_ and liquid biomethane production. SCO_2_—solid carbon dioxide.

**Figure 6:**
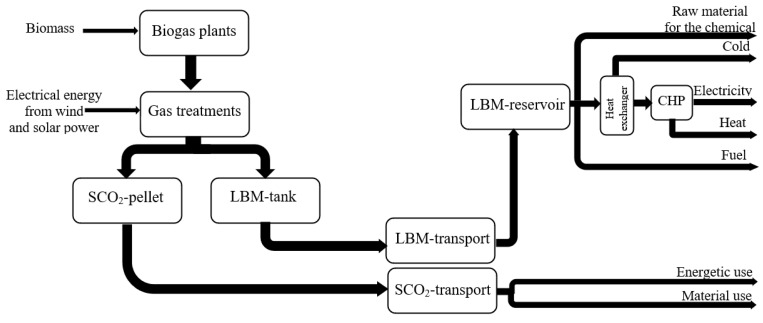
Potential applications of SCO_2_ and liquid biomethane. CHP—combined heat and power; LBM—liquefied biomethane; and SCO_2_—solid carbon dioxide.

One promising approach worth considering is based on the removal of impurities and a pressure-free cryogenic liquefaction unit that converts crude gas from the biogas plant into industrial LBM and industrial SCO_2_ [195]. In the first stage, the crude biogas is converted into a near-pure mixture of methane and carbon dioxide. The gas-cleaning process begins with desulfurization by ferric chloride or other means. This is followed by ammonia removal (e.g., in the form of ammonium sulfate) and fine desulfurization (for example, by way of adsorption in activated carbon columns) [196]. The gas is dried by a gas cooler (7 °C), then given an additional pass through a silica gel and/or zeolite column. In the next stage, the biogas is precooled by a heat exchanger dissolved in alcohol (ethanol or methanol). The exchanger also works as a backup unit by freezing out any leftover gas impurities not removed by the previous step. The process is particularly effective for constituents, such as H_2_O or NH_3_ [197]. A heat exchanger can theoretically operate with dry ice dissolved in alcohol (ethanol or methanol). Approximately 20% of the resulting SCO_2_ has to be appropriated for precooling. The temperature of the gas at this point is around −80 °C. Methane (CH_4_) and carbon dioxide (CO_2_) are separated in the third stage. CO_2_ is specifically flocculated from the gas stream by another heat exchanger that further reduces the temperature and forms the core of the purification system. SCO_2_ can be removed at the base of the heat exchanger setup. The biogas upgrading process with concurrent SCO_2_ and liquid biomethane production is illustrated in Figure 5. Potential applications of these products are listed in Figure 6. 

Of course, the individual steps of the biogas purification/upgrading + SCO_2_ production process must be reselected on an individual basis, depending on the qualitative composition of the crude biogas. The levels of impurities (including H_2_S, NH_3_, and H_2_O) are determined by the original feedstock and the choice of AD technology. The efficient and effective removal of impurities from biogas is a key factor in determining the purity, area of application, and resultant commercial value of the recovered SCO_2_. Dry ice used in the food industry must be high-grade and high-purity [198]. The vapor pressure curves of CO_2_ and H_2_S intersect at such a point that even the most minute remnants of H_2_S in the treated gas will be drawn into the SCO_2_ during cooling. As H_2_S is highly toxic and has a very low odor detection threshold, the purification process must ensure near-complete removal of H_2_S [199]. 

However, it should be stressed that pursuing extremely high-grade SCO_2_ significantly drives up processing costs, which may undermine the economic viability of the process. There is therefore a real need to look for cheaper solutions or other areas of use that could accommodate the lower purity of SCO_2_ produced at biogas plants. One promising avenue could be to use such SCO_2_ for pretreatment that could improve biogas production performance and streamline the purification of biogas prior to SCO_2_ production. 

## 5. Applications of SCO_2_ in Sludge Management

There has been a large number of studies demonstrating that successive freezing and thawing of sludge can produce positive outcomes as part of a sludge pretreatment process [54,200]. In particular, such processing drastically improves sludge dewaterability [201] and reduces the values of respective indices, such as capillary suction time (CST) and resistivity (R). Low-temperature treatments have been found to transform activated sludge flocs into a more compact form, weaken forces, reduce sludge water, facilitate the destruction of cellular structures, and promote the release of intracellular water [202]. The process is promising and offers very good performance in terms of reducing the water content in the final sludge, thus directly reducing volume, storage areas, warehousing requirements, and transport costs for final sludge neutralization and management. 

The rapid drop in temperature causes a heat shock response, which directly affects the cellular structures of the microorganisms, first the external, then the internal ones [203]. The frozen microbial cells are partially destroyed mechanically, which carries a number of positive implications for subsequent sludge treatment [204]. Frozen microbial cells are damaged by the ice crystal forming outside and inside [205]. This has multiple effects, including increased concentration of disintegrated microbes, resultant dissociation of cellular lipoproteins and other organic matter, dissolution of intracellular substances, and release of bound water into the medium [206]. In the next stage of the SCO_2_ process, the medium and the sludge are gradually heated, which, according to the laws of physics, increases the volume of water from the previously formed ice crystals. This drives further disintegration of bacterial cells and improves the efficiency of the subsequent sludge treatment [67]. Thermal disintegration (flash freezing followed by gradual thawing) destroys activated sludge flocs, promotes biomass fragmentation, facilitates efficient dispersion of molecular associations, modifies cell morphology, and denatures macromolecules [207]. The freezing/thawing of the sludge with SCO_2_ triggers effective lysis of microbial cells and thus increases dissolved organic matter [53]. These mechanisms can be harnessed to sanitize and dewater sludge, as well as to pretreat the sludge biomass prior to aerobic or anaerobic stabilization. The process optimizes the subsequent multistage treatment and final neutralization and/or safe management of the sludge [208].

**Figure 7:**
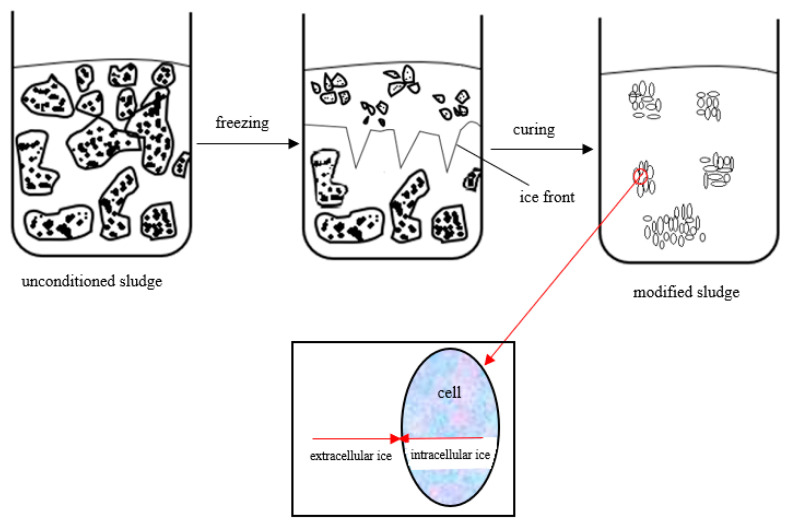
Effect of freezing/thawing on sludge microbe cells.

The specifics and ultimate performance of the freeze/thaw process are determined by the morphology of the cell system, the taxonomic make-up of the microbes, and the diversity in the sludge biomass. The nucleation and growth of ice crystals are also influenced by differences in water volume and activity within cellular structures, nucleation sites, viscosity, membrane permeability, and other factors [209]. A diagram of the freeze/thaw process in sludge microbe cells is presented in Figure 7 [210].

The SCO_2_-induced destruction of microbial cell structures results in an increase in dissolved indicator substances, such as proteins, molecular material, orthophosphates, ammoniacal nitrogen, and carbohydrates, as well as soluble COD [151]. This increases the turbidity of supernatant and decreases CST, which may indicate good dewaterability [133]. An FTIR analysis confirmed the hypothesis that the process is effective at disintegrating sludge, as supported by absorbance changes at specific wavelengths (which corresponded to the presence of amines, amino acids, proteins, phosphates, and other substances). The separation of these substances in the supernatant indicates that the process was destructive to microorganisms and triggered effective lysis of microorganisms cells [67]. Sludge conditioning with SCO_2_ is unquestionably an environmentally friendly technology [208], especially when taking into account that SCO_2_ can be recovered through biogas upgrading and conditioning [211]. The method also avoids the secondary impurities often introduced by chemical disintegration and the additional energy inputs required by mechanical treatment [53].

Nevertheless, freeze/thaw methods have yet to gain much popularity and widespread large-scale use [212]. This lack of interest is mainly attributable to the low cost-effectiveness of the process and high costs of the mechanical freezing [213]. However, SCO_2_ production and use may still prove to be a sustainable alternative, given the current push toward circular economies and the reduction in carbon dioxide emissions [214]. Employing solid CO_2_ in sludge treatment will also reduce the costs of manufacturing the substance from flue gas, waste gas, or biogas (by avoiding stringent purity standards for the end product). 

The freezing process involves extracting heat from the product to a temperature below its crystallization temperature [215]. Thus, analyzing heat transfers in SCO_2_-conditioned sludge is a matter of priority. When analyzing the heat generated by the phase transition of sludge subjected to freezing, factors that must be taken into account include SCO_2_ sublimation, water solidification, heat transferred by other substances, and heat recovered from the sludge [67]. Studies to date have looked at how much heat is transferred between SCO_2_ and sludge when the two are mixed at different ratios, namely 0.25:1, 0.5:1, 0.75:1, and 1:1 [67]. SCO_2_ mixed with sludge at 0.25:1 cooled down to 273.15 K, whereas a ratio of 0.5:1 produced a higher temperature of 267.15 K. At ratios of 0.75:1 and 1:1, the SCO_2_ absorbed 558 kJ and 745 kJ heat, respectively [67].

One way to incorporate SCO_2_ into sludge treatment processes is to use it for the conditioning of pollutants commonly generated by wastewater treatment plants. A study tested the effect of SCO_2_ conditioning on improving the dewatering parameters of four types of sludge: primary sludge, surplus sludge, chemical sludge, and postflotation fatty sludge [133]. The results indicate that SCO_2_ significantly enhances sludge dewaterability. Zone-settling velocity dropped to a range of 76–150 μm/s, and capillary suction time (CST) fell to less than 36 s after conditioning. There was also a significant improvement in sludge dewaterability indicators [133].

The effectiveness of sludge treatment with SCO_2_ has also been demonstrated using biochemical parameters. The best-performing variants produced an over 14-fold increase in the soluble COD, 5-fold increase in total nitrogen and protein, 7-fold increase in carbohydrates, 23-fold increase in ammoniacal nitrogen, and a 27-fold increase in orthophosphates. Furthermore, there was also a significant (more than 7×) increase in the turbidity of the overlying liquid and a 2.23 mg L^−1^ increase in molecular material (RNA) levels [67]. Sewage sludge pretreatment with SCO_2_ was shown to be a promising and sustainable alternative to conventional conditioning, with a 48% improvement in organic matter removal compared to raw (nontreated) biomass [67].

Another study aimed to assess whether SCO_2_ could be used to sanitize waste-activated sludge (WAS). The study compared the commonly used hydrodynamic disintegration with the freeze/thaw method [97]. A microbiological and parasitological analysis showed a significant decrease in pathogenic bacteria, coliphages, and parasite eggs in the disintegrated sludge. The counts of the investigated bacteria (*Salmonella* sp., *Escherichia coli*, and *Clostridium perfringens*) and coliphages were reduced by 19.3–42.3% after hydrodynamic cavitation. By comparison, freezing/thawing with SCO_2_ destroyed between 7.8 and 14.9% of the microbes. The reduction in parasite egg counts (*Ascaris* sp., *Trichuris* sp., and *Toxocara* sp.) for these disintegration methods ranged from 10.7 to 29.3%. The combination of hydrodynamic cavitation and SCO_2_ disintegration synergized well and produced the best results. *Salmonella* sp., *E. coli*, *Clostridium perfringens*, and coliphages in 1 g dry mass decreased by 69.7%, 70.0%, 38.4%, and 48.2%, respectively [97]. The disruption of WAS by a hybrid method reduced the egg numbers for *Ascaris* sp. (63.8%), *Trichuris* sp. (64.3%), and *Toxocara* sp. (66.4%) [97]. 

Frozen microbes, including those frozen by SCO_2_ freezing, die due to volumetric expansion of the freezing water in the cytoplasm, mechanical damage to the cell wall and membrane, osmotic shock, and the destruction of cellular organelles [216]. Mechanical damage is also caused by the formation of ice crystals in the environment within and without the cells, as well as by the partial loss of hydration water of proteins, leading to changes in protein properties. The extracellular crystals, which expand due to freezing, destroy the microbial cells in between [217]. The formation of intercellular crystals leads to damage to the biomembranes and changes their properties, which causes intracellular substances to escape into the environment. Given the characteristics and structure of sewage sludge, as well as the available literature data, LTC-SCO_2_ may offer a technologically and energetically viable alternative to other methods [151,218]. The effects of SCO_2_ conditioning on the physicochemical, biochemical, and sanitary indicators for sewage sludge are listed in Table 6. 

## 6. Applications of SCO_2_ in Sludge Pretreatment 

AD is shaped by the phase transitions that occur during the process. The degree of sludge biodegradation depends on the efficiency of the hydrolysis phase. Surplus sludge, a flocculated suspension of microorganisms, has limited biodegradability in AD and tends to contain large amounts of volatile suspended solids (around 65–75%). 

The effect of freezing/thawing on AD of household and industrial sewage sludge has been investigated by Montusiewicz et al. (2010) [219], Wang et al. (1995) [220], Jan et al. (2008) [221], and Meyer et al. (2017) [222]. Montusiewicz et al. (2010) [219] pretreated a 60:40 mixture of primary sludge and biosludge at −25 °C before digestion. Though the biogas yield as expressed in mL/gVS added and volatile solid (VS) removal did not change, the biogas yield expressed in mL/gVS removed was 1.5 times greater. On average, the soluble COD (sCOD) doubled after the freeze–thaw treatment. Wang et al. (1995) [220] noted a 27% increase in methane production after applying the treatment to municipal biosludge at −10 °C. In turn, Jan et al. (2008) [221] used the freeze/thaw method on bakery biosludge at −17 °C, then digested it anaerobically for 25 days. The COD removal for this process was 30%, compared to 18% in the nonpretreated sludge. The positive effect of freezing/thawing on AD performance was also corroborated by Meyer et al. (2017) [222], who tested its impact on the dewatering and AD of pulp/paper mill sludge. The treatment was more successful in improving the dewaterability of mill biosludge samples than dewatering with polymer. Treatment at −10 °C prior to dewatering increased the dry matter content of the dewatered digestate from 10% to 20% (after 35-day digestion) and from 17% to 23% (after 60-day digestion). The specific biogas yield increased from 111 to 310 cm^3^·g^−1^ chemical oxygen demand added [222]. The thermal treatment of sludges with SCO_2_ shows promise in improving digestion performance. As the surplus sludge is disintegrated by SCO_2_, the microbial cells are denatured through their scaffold structure. Crystallization commences, and surplus sludge microbes undergo what is known as a “heat shock response”.

A study by Kazimierowicz et al. (2020) [151] investigated how the low-temperature pretreatment of dairy sewage sludge with SCO_2_ affects AD performance. Increasing the SCO_2_-to-sludge by volume ratio beyond 0.3 did not produce significant changes in the soluble chemical oxygen demand. The highest COD values ranged from 490.6 ± 12.9 to 510.5 ± 28.5 mg·dm^−3^, whereas nonconditioned sludge contained 400.5 ± 23.8 mg·dm^−3^. Low-temperature conditioning increased the levels of ammoniacal nitrogen from 155.2 ± 10.2 to 185.9 ± 11.1 mg·dm^−3^, whereas orthophosphates increased from 198.5 ± 23.1 to 300.6 ± 35.9 mg·dm^−3^. The peak value of the specific biogas yield was 630.2 ± 45.5 cm^3^·g DM^−1^, obtained at a 0.3 ratio of SCO_2_-to-dairy sewage sludge (by volume). The methane fraction in the biogas was approx. 68.7 ± 1.5% [151]. Increased SCO_2_ did not produce significant changes in biogas or methane production. The efficiency of biogas production from nonconditioned dairy sludge was lower by 43.0 ± 3.2%. The experiment showed a very strong positive correlation between concentrations of dissolved COD, N-NH^4+^, and P-PO_4_^3−^ and biogas yield at SCO_2_/dairy sewage sludge (DSS) ranges between 0 and 0.3% The energy performance analysis demonstrated that LTC-SCO_2_ is an energy-efficient technology. Peak net energy production was 32.3 ± 1.5 Wh/dm^3^ DSS. This method yielded 13% more energy that the nonconditioned DSS variant [151].

Another study compared the performance of SCO_2_-treated surplus sludge vs. nontreated surplus sludge [208]. Again, the experiment demonstrated that the modified sludge had higher biodegradability in anaerobic conditions. Hydrolysis was found to begin as early as the thermal treatment stage, along with the corresponding increases in indicators, such as soluble chemical oxygen demand (SCOD), volatile fatty acids (VFAs), and total organic carbon (TOC). The SCO_2_ treatment produced the best results at 0.35:1 SCO_2_-to-surplus sludge ratio by volume. The degree of sludge disintegration (6.6%) also pointed to this variant as the optimal option. The SCO_2_-disintegrated sludge (at the optimal reagent dose) had higher levels of SCOD, TOC, and VFA than the nontreated surplus sludge throughout the digestion process [208].

Another study aimed to assess how disintegration by SCO_2_ affects AD of modified surplus sludge [53]. SCO_2_ pellets (0.6 mm in diameter) were used as the treatment reagent. The SCO_2_ was mixed with surplus sludge at ratios of 0.15/1, 0.25/1, 0.35/1, 0.45/1, 0.55/1, 0.65/1, and 0.75/1 by volume. The AD process was run for 8 and 28 days under mesophilic conditions at 37 °C. Untreated sludge was used in the first series. The second and third series used the following treatment parameters: proportion of the SCO_2_ dose to sludge (by volume): 0.55/1; pretreatment time: 12 h. Sludge disintegration, percentage of sludge digested (digestion degree), and biogas yield improved, indicating that the treatment had a positive effect. The best results were obtained at a reagent-to-surplus sludge ratio of 0.55/1 (by volume). The optimal treatment parameters led to 2.7-, 3-, and 2.8-fold increases in the TOC, SCOD, and VFA levels, respectively, against the nontreated variant. The percentage of sludge digested and the biogas yield were 33% and 31% higher, respectively, than in the nontreated sludge [53].

There have also been investigations into activated sludge disintegration prior to thermophilic anaerobic stabilization. In one example, sewage sludge was subjected to a combined chemical + thermal pretreatment with NaOH and SCO_2_ [223]. The treatment was found to improve organic removal and anaerobic stabilization during digestion compared with raw sludge. The experiment utilized a hybrid process that began with alkalization to 9.5 pH, after which the sludge was conditioned with SCO_2_ at a 1:1 ratio by volume [223]. The combination proved to have a synergistic effect and led to greater disruption/disintegration of microbial biomass and sludge flocs. The synergistic action restored the pH after alkalization and promoted the release of soluble organic matter (the SCOD was 2000 mg·L^−1^ higher for the hybrid process). Improved biogas yield and production were noted after thermophilic digestion [223]. As the volume of disintegrated WAS in the digester increased, so did biogas production. Improved biogas production (approximately 59% higher in comparison to the blank trial) and biogas yields (approximately 31% higher in comparison to the blank trial) were obtained at 50% WAS by volume. The recorded biogas production and yields after 21-day digestion were 26.6% and 2.7% higher, respectively, than in the blank trial. Subjecting the sludge to the hybrid process before anaerobic stabilization also led to better sanitization [223].

These findings are corroborated by another experiment, which tested how a hybrid disintegration process with alkalization (pH ≈ 9) and freezing/thawing with SCO_2_ (1:0.75 SCO_2_-to-sludge ratio by volume) affects surplus-activated sludge and mesophilic AD [224]. The study found that the COD of nondisintegrated surplus sludge averaged 100 mg/dm^3^, whereas subjecting the activated sludge to the combined chemical + thermal disintegration treatment led to the organic matter in overlying liquid (expressed by the difference in soluble chemical oxygen demand—COD) rising to approx. 1890 mg/dm^3^. Harnessing and feeding the disintegrated sludge into digesters at different ratios produced various effects on the biogas production and yield. The 50%-disintegrated-sludge batch benefited the most in terms of biogas production compared to the other samples at 2.933 dm^3^ (15.2% increase), whereas the yield was the highest in the 30%-disintegrated-sludge sample at 0.482 dm^3^/gVS_removed_. The hybrid disintegration process is simple, easy to implement in full-scale plants, and does not affect the pH of the input sludge (SCO_2_ neutralizes previously alkaline sludge) [224]. A performance comparison of SCO_2_-based sludge pretreatment processes is provided in Table 7. 

## 7. Estimated Energy and Economic Efficiency

Reliable results of energy and economic analyses can be obtained based on research work carried out using installations operated on a fractional–technical and pilot scale. Only in this case does long-term exploitation work allow to collect the appropriate amount of data and determine the source and size of the variability of the obtained results [225]. Complex technologies based on many unit processes, including sustainable production and use of SCO_2_ in sewage sludge management, mainly in anaerobic digestion, require a high technological readiness level (TRL) and comprehensive research. They should aim at determining the flows of energy and matter, as well as at a comprehensive assessment of the environmental impact, including the real carbon footprint, while taking into account investment and operating costs as well as possible revenues [226]. It is necessary to perform an environmental Life Cycle Costing (LCC) analysis and Life Cycle Assessment (LCA) analysis [227].

At present, only estimations can be made based on input data, predictions, and results of experimental work carried out on a laboratory scale. This has been identified in studies [151]. During the anaerobic digestion of sewage sludge without the use of SCO_2_, the amount of CH_4_ obtained was 270 dm^3^/kg VS. The use of pretreatment using SCO_2_ allowed to increase the CH_4_ efficiency in the range from 379 to 434 dm^3^/kg VS, depending on the dose of the SCO_2_ used. The energy analysis characterizes the production of SCO_2_ from pure CO_2_ in a commercial installation available on the market. Considering the energy demand of the analyzed SCO_2_ generator, it was proven that it is possible to obtain a positive energy balance in several tested technological variants of up to 3.0 ± 1.0% and to 13.1 ± 1.1%. The net energy gain ranged from 28.6 ± 1.5 kWh/Mg of sewage sludge in the variant without the use of SCO_2_ to 32.3 ± 1.5 kWh/Mg of sludge for the SCO_2_/sludge volume ratio of 0.3 [151].

This kind of pretreatment to intensify the anaerobic digestion of sewage sludge could be even more justified if a closed CO_2_ cycle was used, including biogas production–biogas enrichment–SCO_2_ production–sludge disintegration–fermentation–biogas production. This is an important argument that improves the economic and technological efficiency of fermentation processes and responds to the reduction in CO_2_ emissions into the atmosphere, which is necessary from the point of view of environmental protection. Taking into account the average prices Carbon Permits (CPPs) in EUR/MgCO_2_ from 2020–2022, based on the data provided by Trading Economics [228] and the amounts of biogas produced in various variants of SCO_2_ application, it was assessed that additional revenue from reducing CO_2_ emissions is possible up to EUR 100/MgTS.

## 8. Conclusions

Operation of sewage treatment plants inevitably leads to the production of sludge. Due to the composition and characteristics of sludge, it must be neutralized and managed through processing. Though numerous methods to that end have been tested and verified in experimental studies and in practice, technologically and commercially competitive solutions still need to be sought.

One promising proposal calls for harnessing SCO_2_ to process sewage sludge. Solid CO_2_ is a normal byproduct of natural gas treatment processes and can also be produced by dedicated biogas upgrading technologies. Given the origin and sourcing of SCO_2_, this method could be considered material recycling and is fully in line with the principles of the circular economy. The technology can also help limit carbon dioxide emissions by sequestering and feeding it into a closed-loop process. Producing and using SCO_2_ in sludge disintegration processes encompasses the capture, extraction, transport, and long-term storage of CO_2_ in a suitable and safe location. 

To date, little information has been reported in the world literature regarding the feasibility of low-temperature conditioning of excess sludge using solidified carbon dioxide (LTC-SCO_2_), meaning that it is still a relatively nascent technology. Sludge conditioning with SCO_2_ is unquestionably an environmentally friendly approach, especially because SCO_2_ can be recovered through biogas upgrading and conditioning. The method also avoids the secondary impurities often introduced by chemical disintegration and the additional energy inputs required by mechanical treatment.

The SCO_2_ conditioning of sludge triggers effective lysis of microbial cells, which destroys activated sludge flocs, promotes biomass fragmentation, facilitates efficient dispersion of molecular associations, modifies cell morphology, and denatures macromolecules. This results in increased levels of dissolved organic matter, nutrients, and molecular material. These mechanisms can be harnessed to sanitize and dewater sludge, as well as to pretreat sludge biomass prior to aerobic or anaerobic stabilization. Sludge processed this way is easier to treat in the subsequent stages and to neutralize and/or manage safely.

Given the presented advantages of using SCO_2_ to process sludge, it can be used as an attractive pretreatment tool to improve methane digestion and fermentative hydrogen production. Furthermore, it can also be incorporated into a closed CO_2_ cycle of biogas production–biogas upgrading–SCO_2_ production–sludge disintegration–digestion–biogas production. This feature not only bolsters the technology’s capacity to improve the performance and cost-effectiveness of digestion processes, but can also help reduce atmospheric CO_2_ emissions, a crucial advantage in terms of environment protection.

This new approach to SCO_2_ production and application largely counteracts previous limitations, which are mainly related to the low cost-effectiveness of the production process. Harnessing SCO_2_ for sludge processing may prove to be an increasingly attractive alternative to other methods, given the current push toward circular economies and reducing carbon dioxide emissions.

## Figures and Tables

**Table 1:** Characteristics and composition of sewage sludges.

Parameter	Unit	Primary Sludge			Secondary Sludge			
Total dry solids	weight as per dry basis (wt.%)	5–9	27.58	27.58	0.8–1.2	25.36	25.36	0.83–12
Volatile solids		60–80	60–80	18.8	59–68	59–68	15.5	30.88
Phosphorous		0.8–2.8		34.13	0.5–0.7		28.76	0.8–11
Nitrogen		1.5–4	33.82	33.82	2.4–5.0	49.91	49.91	1.5–6
Protein		20–30	2–30		33–41	32–41		15–41
Lipids		7–35			5–12			
Potassium		0–1			0.5–0.7			0.4–3
Cellulose		8–15			7–9.7			
Silica		15–20						10–20
Iron	Fe g/kg	2–4						
pH	-	5–8		5.61	6.5–8		6.42	5–8
Organic acids	mg/L as acetate	200–2000			1100–1700			200–2000
Alkalinity	mg/L as CaCO_3_	500–1500			580–1100			
Energy content	kJ/kg DM	23,000–29,000	23,000–29,000		19,000–23,000	19,000–23,000		
Reference:		[83]	[84]	[85]	[83]	[84]	[85]	[86]
Parameter	Unit	Sewage sludge						
Moisture content	weight as per dry basis (wt.%)	7.65 ^a^	6.1 ^a^	10.84 ^a^	7.33 ^a^	2.37 ^a^	1.05 ^a^	78 ^b^
Volatile matter content		51.66 ^a^	53.0 ^a^	48.31 ^a^	62.97 ^a^	45.47 ^a^	47.92 ^a^	55.8 ^e^
Ash content		35.02 ^a^	38.4 ^a^	33.88 ^a^	16.33 ^a^	45.81 ^a^	45.51 ^a^	33.7 ^e^
Fixed carbon		5.67 ^b^	8.6 ^a^	6.97 ^b^	13.37 ^b^	6.35 ^b^	5.52 ^b^	10.5 ^e^
Higher Heating Value	MJ/kg	13.16	13.9	11.79	15.2	11.14	-	-
C	weight as per dry basis (wt.%)	58.5 ^c^	31.1 ^a^	27.38 ^d^	38.28 ^d^	24.63 ^a^	25.93 ^a^	32.8 ^a^
H		5.8 ^c^	4.2 ^a^	3.92 ^d^	5.92 ^d^	3.32 ^a^	4.21 ^a^	10.2 ^a^
N		0.53 ^c^	3.3 ^a^	9.90 ^d^	1.0 ^d^	2.96 ^a^	4.78 ^a^	5.4 ^e^
S		1.43 ^c^	1.1 ^a^	0.45 ^d^	0.09 ^d^	1.06 ^a^	1.03 ^a^	1.7 ^a^
O		33.74 ^b^	24.3 ^a^	13.64 ^b^	31.06 ^b^	19.85 ^b^	22.02 ^a^	24.5 ^a^
Reference:		[87]	[88]	[89]	[90]	[91]	[92]	[93]

a = dry basis, b = by difference, c = dry ash-free basis, d = as-received, and e = moisture-free basis.

**Table 2:** Concentrations of heavy metals in sewage sludge.

Element	Concentration	Permitted Range	Ref.
	mg/kg DM		
As	5.6–56.1	not limited	[10,98,99]
Ba	41.5–1300	not limited	[10,98,99]
Cd	0.83 ± 0.06	20–40	[98,99,100]
Cr	18.6 ± 2.2	not limited	[98,99,100]
Cu	75.8 ± 7.0	1000–1750	[10,99,100]
Hg	0.1–1.1	16–25	[10,99]
Mo	1.7–75	not limited	[10,99,101]
Ni	8.6–420	300–400	[10,99,101]
Pb	4.0–429.8	750–1200	[10,99,101]
Se	2	not limited	[10,99,101]
Zn	0–7500	2500–4000	[10,99]

**Table 3:** Sanitary indicators for municipal sewage sludge.

Type	Organism	Density					
		#/g DM		Bacteria/g DM		cfus/g	MPN/g DM
Virus	Various enteric viruses	10^2^–10^4^	3·10^2^			87–417·10^7^	
Bacteria	Total coliforms	10^8^–10^9^	7·10^8^	10^4^–10^9^	1.1·10^9^		3.2·10^9^
	Fecal coliforms	10^7^–10^8^	8·10^6^	10^4^–10^8^	1.9·10^5^		3·10^7^
	Fecal streptococci	10^6^–10^7^	2·10^2^				
	*Salmonella* sp.	10^2^–10^3^	9·10^2^	10^3^–10^6^	2.9·10^2^		3.3·10^7^
Protozoa	*Giardia* sp.	10^2^–10^3^	10^2^–10^3^				
Helminths	*Ascaris* sp.	10^2^–10^3^	1·10^3^			1.75	
	*Trichuris vulpis*	10^2^	<10^2^				
	*Toxocara* sp.	10^1^–10^2^	3·10^2^			3.25	
Reference:		[102,103] *	[102,103] **	[104,105]	[105,106]	[98,107,108,109]	[110]

cfus—colony-forming units; MPN—most probable number; *—primary sludge; and **—secondary sludge.

**Table 4:** Organic micropollutants in sludges.

Compound		Concentration ng/g	Ref.
PAHs	Anthracene	13–724	[98,118]
	Benzofluoranthene	9.9–1477	[98,118]
	Benzopyrene	17.9–1475.5	[10,98]
	Chrysene	21–2020.5	[98,118]
	Fluoranthene	34.5–3216.8	[98,118]
	Phenanthrene	13–5552.2	[98,118]
	Pyrene	47.2–26,337	[98,118]
PhCs found in sewage sludge-amended soils	Caffeine	not detected	[10,119,120]
	Ciprofloxacin	350–400	[10,119,120]
	Diclofenac	1.16	[10,119,120]
	Galaxolide	633	[10,119,120]
	Ibuprofen	5.03	[10,119,120]
	Triclosan	833	[10,119,120]
	Trimethoprim	0.64	[10,119,120]
	Tonalide	113	[10,119,120]
PFASs	PFOA	1.22	[10,121]
	PFOA	1	[10,122]
	PFOS	1.31	[10,121]
	PFOS	5	[10,122]

**Table 5:** Characteristics of SCO_2_.

Type of SCO_2_	Size	Appearance	Sublimation Rate	Primary Users	Application	Ref.
Snow	N/A	Similar to water snow	Fastest sublimation rate; Shortest shelf life; Quick cooling	Meat establishments	Flash freezing	[135,145,150]
Pellets	1–3 mm	Rice-like granules	Fast sublimation rate; Short shelf life; Quick cooling	Processors of foodstuffs; DI blasting companies; Theaters and nightclubs; Farmers; Fire services; Car mechanics	Short-distance, small-parcel shipping Dry ice blasting; Food processing (for freezing foodstuffs); Smoke and fog effects for theaters and nightclubs; Rodent control; Firefighting; Automotive mechanics; Sludge management and pretreatment	[135,148,150,151]
Nuggets	6–19 mm	Small cylinders of dry ice	Average sublimation rate; Average shelf life	Bioservices companies (laboratories); Bakeries; Meat establishments	Long-distance, large-parcel shipping; Food processing (for packing and shipping foodstuffs/products) Sludge management and pretreatment	[135,147,150]
Slabs	210 × 125 × 18 mm (standard block dimensions vary by country)	Strips or boards of dry ice	Slow sublimation rate	Long shelf life Distributors; Airline caterers	Shipping; Airline catering (a typical 19 mm strip matches the size of catering trays); Corpse refrigeration	[135,145,150]
Blocks	250 × 250 × 125 mm (standard block dimensions vary by country)	Blocks of dry ice	Slowest sublimation rate	Longest shelf life Grocery store warehouses; Ice cream parlours	Shipping; Shaved ice blasting; Food processing; Corpse refrigeration	[135,148,150]

**Table 6:** Effect of conditioning with SCO_2_ on sludge.

Sewage Sludge	Sewage Sludge before Conditioning	SCO_2_-to-Sludge Ratio (by Volume)	Effect of Conditioning	Ref.
Waste-activated sludge	SCOD: 65 mg/L; Proteins: 56 mg/L; RNA: 10.07 mg/L; Carbohydrates: 12 mg/L; Ammoniacal nitrogen: 1.1 mg/L; Phosphates: 48 mg/L; Capillary suction time (CST): 46.2 s Turbidity: 57 mg SO_2_/L	0.25/1	SCOD: 205 mg/L; Degree of disintegration: 15% Proteins: 99 mg/L; RNA: 10.35 mg/L; Carbohydrates: 27 mg/L; Ammoniacal nitrogen: 8.5 mg/L; Phosphates: 52 mg/L; CST: 44.9 s Turbidity: 274 mg SO_2_/L	[67]
		0.50/1	SCOD: 480 mg/L; Degree of disintegration: 28% Proteins: 155 mg/L; RNA: 11.0 mg/L; Carbohydrates: 39 mg/L; Ammoniacal nitrogen: 15.5 mg/L; Phosphates: 98 mg/L; CST: 34.8 s Turbidity: 310 mg SO_2_/L	
		0.75/1	SCOD: 600 mg/L; Degree of disintegration: 39% Proteins: 200 mg/L; RNA: 11.95 mg/L; Carbohydrates: 50 mg/L; Ammoniacal nitrogen: 18.8 mg/L; Phosphates: 122 mg/L; CST: 28.5 s Turbidity: 370 mg SO_2_/L	
		1/1	SCOD: 889 mg/L; Degree of disintegration: 48% Proteins: 291 mg/L; RNA: 12.23 mg/L; Carbohydrates: 83 mg/L; Ammoniacal nitrogen: 24.0 mg/L; Phosphates: 133 mg/L; CST: 22.8 s Turbidity: 410 mg SO_2_/L	
Waste-activated sludge	CST: 43.7 s Solid content: 6.25 % *w*/*w* Index for the bound moisture and structure of the sludge flocs (*h*_f_/*h*_I_): 0.97	75 g/200 g	Zone settling velocity (ZSV): 76.1 μm/s CST: 33.9 s Solid content: 15.7 % *w*/*w* *h*_f_/*h*_I_: 0.35	[133]
Ferric hydroxide sludge	ZSV: 94 μm/s CST: 51.8 s Solid content: 14.5 % *w*/*w* *h*_f_/*h*_I_: 0.35 Particle size: 61.7 μm		ZSV: 390 μm/s CST: 38.7 s Solid content: 18.6 % *w*/*w* *h*_f_/*h*_I_: 0.096 Particle size: 51.8 μm	
Oily sludge	CST: 87.2 s Particle size: 15.5 μm		CST: 58.7 s Particle size: 26.2 μm	
Waste-activated sludge	*E. coli*: 5.88 log cfus/gTS; *Ascaris* sp.: 2.08 log eggs/kgTS; *Trichuris* sp.: 1.96 log eggs/kgTS; *Toxocara* sp: 3.05 log eggs/kgTS;	1/1	*E. coli*: 5.82 log cfus/gTS; *Ascaris* sp.: 2.03 log eggs/kgTS; *Trichuris* sp.: 1.88 log eggs/kgTS; *Toxocara* sp: 2.34 log eggs/kgTS;	[97]

**Table 7:** Performance of SCO_2_-based sludge pretreatment processes.

Sewage Sludge	Sewage Sludge before Pretreatment Processes	SCO_2_-to-Sludge Ratio (by Volume)	Performance of SCO_2_-Based Sludge Pretreatment Processes	Ref.
Waste-activated sludge	TS: 10.89 ± 0.27 * g/L; VSS: 7.05 ± 0.75 * g/L; VFAs: 75 ± 4 mg CH_3_COOH/L, 238 ± 2.4 * mg CH_3_COOH/L; SCOD: 126 ± 4 mg O_2_/L, 561 ± 3.7 * mg O_2_/L; TOC: 42 ± 1 mg/L, 193 ± 1.5 * mg/L; Kjeldahl nitrogen: 56 ± 2 mg N/L, 965 ± 2.5 * mg N/L; Ammoniacal nitrogen: 52 ± 1 mg N-NH_4_/L, 941 ± 4.7 * mg N-NH_4_/L; pH: 7.2 ± 0.1, 7.14 ± 0.15 *; Alkalinity: 3120 ± 10 * mg Ca CO_3_/L; Digestion degree: 40%; Biogas: 0.43 L/gVSS	0.55/1	TS: 7.94 ± 0.64 * g/L; VSS: 4.55 ± 0.41 * g/L; VFAs: 245 ± 5 mg CH_3_COOH/L, 321 ± 1.6 * mg CH_3_COOH/L; SCOD: 400 ± 10 mg O_2_/L, 761 ± 7.3 * mg O_2_/L; TOC: 110 ± 2 mg/L, 211 ± 1.2 * mg/L; Kjeldahl nitrogen: 78 ± 2.5 mg N/L, 995 ± 2.7 * mg N/L; Ammoniacal nitrogen: 90 ± 2 mg N-NH_4_/L, 982 ± 2.4 * mg N-NH_4_/L; pH: 6.4 ± 0.1, 6.87 ± 0.06 *; Alkalinity: 3820 ± 28 * mg Ca CO_3_/L; Digestion degree: 60%; Biogas: 0.62 L/gVSS	[53]
	VFAs: 65 mg CH_3_COOH/L, 519 * mg CH_3_COOH/L; SCOD: 110 mg O_2_/L, 143 * mg O_2_/L; TOC: 26 mg/L, 484 * mg/L; pH: 7.04	0.35/1	VFAs: 164 mg CH_3_COOH/L, 954 * mg CH_3_COOH/L; SCOD: 293 mg O_2_/L, 2731 * mg O_2_/L; TOC: 78 mg/L, 831 * mg/L; pH: 6.35	[208]
	Biogas: 2380 ± 78 mL/L; Methane: 61 ± 1%	1/1 + hydrodynamic cavitation	Biogas: 2622 ± 82–3860 ± 132 mL/L; Methane: 61 ± 1–64 ± 2%	[97]
	SCOD: 123 ± 20 mg O_2_/L; Biogas: 2543 mL/d/L	1/1 +2M NaOH	SCOD: 2120 ± 75 mg O_2_/L; Biogas: 3310–3843 mL/d/L	[223]
	SCOD: 100 ± 4 mg O_2_/L; Biogas: 2547 L; Methane: 59–62%	0.75/1 +2M NaOH	SCOD: 1890 ± 73 mg O_2_/L; Biogas: 2090–2933 L; Methane: 61–64%	[224]
Dairy sewage sludge	SCOD: 400.5 ± 23.8 mg O_2_/L; Ammoniacal nitrogen: 131.5 ± 16.7 mg N-NH_4_/L; Orthophosphate: 159.3 ± 22.4 mg P-PO_4_^3−^/L Biogas: 440.7 ± 21.5 mL/gVS; Methane: 61.2 ± 1.3%	0.1/1	SCOD: 450.3 ± 25.6 mg O_2_/L; Ammoniacal nitrogen: 155.2 ± 10.2 mg N-NH_4_/L; Orthophosphate: 198.5 ± 23.1 mg P-PO_4_^3−^/L Biogas: 528.84 ± 38.5 mL/gVS; Methane: 63.8 ± 2.8%	[151]
		0.2/1	SCOD: 479.2 ± 10.5 mg O_2_/L; Ammoniacal nitrogen: 166.8 ± 11.4 mg N-NH_4_/L; Orthophosphate: 236.9 ± 25.8 mg P-PO_4_^3−^/L Biogas: 564.10 ± 41.6 mL/gVS; Methane: 64.5 ± 1.7%	
		0.3/1	SCOD: 490.6 ± 12.9 mg O_2_/L; Ammoniacal nitrogen: 171.2 ± 10.5 mg N-NH_4_/L; Orthophosphate: 260.1 ± 20.1 mg P-PO_4_^3−^/L Biogas: 630.20 ± 45.5 mL/gVS; Methane: 68.7 ± 1.5%	
		0.4/1	SCOD: 495.2 ± 26.4 mg O_2_/L; Ammoniacal nitrogen: 180.3 ± 12.6 mg N-NH_4_/L; Orthophosphate: 275.6 ± 33.4 mg P-PO_4_^3−^/L Biogas: 581.72 ± 39.4 mL/gVS; Methane: 66.3 ± 2.1%	
		0.5/1	SCOD: 510.5 ± 28.5 mg O_2_/L; Ammoniacal nitrogen: 185.9 ± 11.1 mg N-NH_4_/L; Orthophosphate: 300.6 ± 35.9 mg P-PO_4_^3−^/L Biogas: 572.91 ± 32.2 mL/gVS; Methane: 66.2 ± 1.9%	

* Digestate assays.

## Data Availability

Not applicable.

## Abbreviations

AD	anaerobic digestion
As	arsenic
Ba	barium
C	carbon
Cd	cadmium
CH_4_	methane
CHP	combined heat and power
cfus	colony-forming units
COD	chemical oxygen demand
CO_2_	carbon dioxide
CPC	Chinese Petroleum Corporation
Cr	chrome
CST	capillary suction time
Cu	copper
DI	dry ice
DM	dry matter
DSS	dairy sewage sludge
EU	European Union
FTIR	Fourier-transform infrared
GHG	greenhouse gas
H	hydrogen
Hg	mercury
H_2_O	hydrogen oxide (water)
H_2_S	hydrogen sulfide
IATA	International Air Transport Association
LBM	liquefied biomethane
LNG	liquid natural gas
LTC-SCO_2_	low-temperature conditioning using solidified carbon dioxide
Mn	manganese
Mo	molybdenum
MPN	most probable number
N	nitrogen
NH_3_	ammonia
Ni	nickel
N/A	not applicable
O	oxygen
P	phosphorus
PAHs	polycyclic aromatic hydrocarbons
Pb	lead
PCBs	polychlorinated biphenyls
PFASs	perfluoroalkyl substances
PFCs	perfluorocarbons
PFOA	perfluorooctanoate
PFOS	perflouroctane sulfonate
PhCs	pharmaceuticals
R	resistivity
RNA	ribonucleic acid
S	sulfur
SCOD	soluble chemical oxygen demand
SCO_2_	solid carbon dioxide
SDG	Sustainable Development Goal
Se	selenium
TOC	total organic carbon
TRL	technology readiness level
UN	United Nations
US	United States
WAS	waste-activated sludge
WWAP	World Water Assessment Programme
VFAs	volatile fatty acids
VS	volatile solid
VSS	volatile suspended solid
Zn	zinc
